# Development and Characterization of Thermal Flow Sensors for Non-Invasive Measurements in HVAC Systems

**DOI:** 10.3390/s19061397

**Published:** 2019-03-21

**Authors:** Samir Cerimovic, Albert Treytl, Thomas Glatzl, Roman Beigelbeck, Franz Keplinger, Thilo Sauter

**Affiliations:** 1 Department for Integrated Sensor Systems, Danube University Krems, A-2700 Wiener Neustadt, Austria; albert.treytl@donau-uni.ac.at (A.T.); thomas.glatzl@donau-uni.ac.at (T.G.); roman.beigelbeck@donau-uni.ac.at (R.B.); thilo.sauter@donau-uni.ac.at (T.S.); 2 Institute of Sensor and Actuator Systems, Vienna University of Technology, A-1040 Vienna, Austria; franz.keplinger@tuwien.ac.at

**Keywords:** thermal flow sensor, non-invasive flow rate measurement, HVAC systems

## Abstract

We investigated non-invasive flow rate measurements in heating, ventilation, and air conditioning (HVAC) systems utilizing thermal transduction instead of commonly used ultrasonic techniques. The proposed thermal flow transduction comprises two temperature sensors and a heater, all mounted non-invasively on the outer surface of metal-pipes and, therefore, not disturbing the fluid flow inside. One temperature sensor measures the heater temperature, whereas the other one, mounted upstream of the heater, follows the fluid temperature for reference. The temperature difference (i.e., the heater excess temperature) depends on the fluid flow velocity and can be used to derive the mean volume flow inside the pipe. Experimental characterizations were conducted using two sensor prototypes. Beside output characteristics, other main issues such as dynamic behavior and noise density were investigated in detail. Special attention was paid to error compensation allowing measurements within a large range of fluid temperatures. Measurement results confirm the feasibility of this approach, however with some constraints regarding response time.

## 1. Introduction

Heating, ventilation, and air conditioning (HVAC) systems contribute significantly to the overall energy consumption of modern buildings. Energy used for heating and cooling obviously has the largest share. Figures vary in different studies, but the share is well above 60–70% of the primary energy used in major parts of the world such as US, EU, China, and Australia [1,2]. Given the enormous energy consumption of HVAC systems, it is evident that their optimal operation can contribute to the overall energy efficiency of a building. Indeed, several studies indicated significant savings potential by improved fault diagnosis and control strategies [3].

In order to optimize the operation of an HVAC system or to detect anomalies, it is essential to monitor the energy flows within the system. To that end, fluid temperature and flow velocity must be obtained at strategically selected points. This applies in particular to heating circuits which have the highest potential for energy savings. Usually, the measurement data in state-of-the-art HVAC systems are collected by means of invasive temperature and flow sensors that have been installed during system setup. However, there is also a high optimization potential in existing building automation systems, which were built years ago and have no respective instrumentation for data acquisition. Such cases require non-invasive sensors that can be clamped on the pipes. This is, first of all, a matter of installation simplicity, but can also be a question of liability, especially when energy analysis is done by third-party service providers that are not allowed to modify hydraulic circuits. Another important example is the buildings under monumental protection, where modifications of an existing hydraulic system are often not possible. Therefore, we started a project dealing with optimizations of building energy efficiency through a model-based energy flow analysis. An important boundary condition thereby was to collect required data by using only non-invasive sensors mounted on metal pipes of HVAC systems.

The most common non-invasive flow sensors are ultrasonic (US) sensors. However, these are sophisticated and very costly devices and, hence, not suited for retrofitting of old HVAC systems on a large scale. Searching for a promising alternative, we have studied the utilization of low-cost thermal flow sensors to hydraulic circuits with metal pipes. According to the underlying physical principle, there are three different types of thermal flow sensors [4,5]:

(1) Hot-wire or hot-film flow sensors, which exploit directly the cooling effect of the passing fluid on a heater. Here, the heater excess temperature (i.e., the difference between heater and fluid temperature) corresponds to the flow velocity [6,7,8].
(2) Calorimetric flow sensors utilize the flow-dependent asymmetry of the temperature profile around the heater. In this case, temperature sensors arranged around the heater are needed for detection. Their temperature difference is a function of the flow velocity [9,10,11].
(3) Time-of-flight (TOF) flow sensors measure the propagation of a heat pulse over a known distance between the heater and the temperature sensor located downstream of the heater [12,13,14].

Owing to high thermal conductivity of metal pipes (compared to PE and PVC pipes), the excess temperature along the pipe surface drops significantly with increasing distance from the heater, even if very high heating power is applied. Hence, calorimetric and TOF sensors are less suitable for non-invasive measurements on metal pipes in HVAC systems. In the course of this feasibility study, we, therefore, concentrated on the hot-wire transduction and developed prototypes, which were tested on common copper pipes. In contrast to sophisticated US setups, this transduction principle suits for arbitrary pipe diameters and allows for an extremely simple, external retrofit implementation.

Deployment of thermal flow sensors in HVAC systems is not broadly considered because of two main reasons. First, a relatively high heating power is needed to achieve reasonable excess temperatures. Secondly, due to the high thermal mass of the sensor system, long response times must be accepted. Therefore, we paid special attention to the thorough transient characterization of sensor prototypes.

## 2. Sensor Design and Operating Principle

Common hot-wire or hot-film flow sensors typically consist of one heater only (in form of a thin wire or film) serving as a heat source and a temperature sensor simultaneously. For this application, we decided to use Pt100 elements as temperature sensors accompanied by wired heaters needed for high heating powers. Those temperature sensors are commercially available in many different forms and feature much higher resistance and faster reactions times compared to wired heaters. The developed sensor prototypes can be characterized as modified hot-film transducers because Pt100 elements are used to evaluate the convective cooling of a heater.

**Figure 1:**
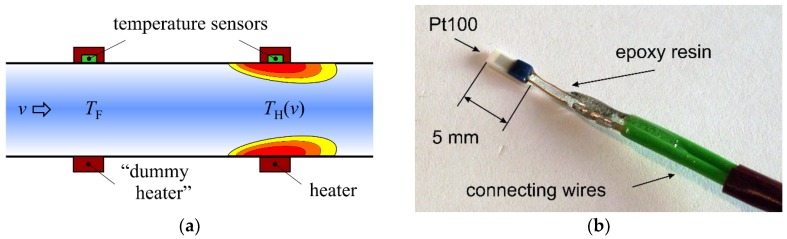
(**a**) Schematic cross-section of the sensor; (**b**) Pt100 elements used for the first sensor prototype.

The basic operating principle can be best explained by means of a schematic cross-section of the sensor depicted in Figure 1a. The sensor consists of a heater and two Pt100 elements which measure the approximate fluid *T*_F_ temperature and the temperature *T*_H_ below the heater. The flowing fluid cools down the area around the heater, and therefore the difference of these temperature values (i.e., the heater excess temperature Δ*T* = *T*_H_ − *T*_F_) correlates with the mean flow velocity *v*. A signal conditioning circuit described in the next section transform the heater excess temperature into an appropriate voltage signal.

**Figure 2:**
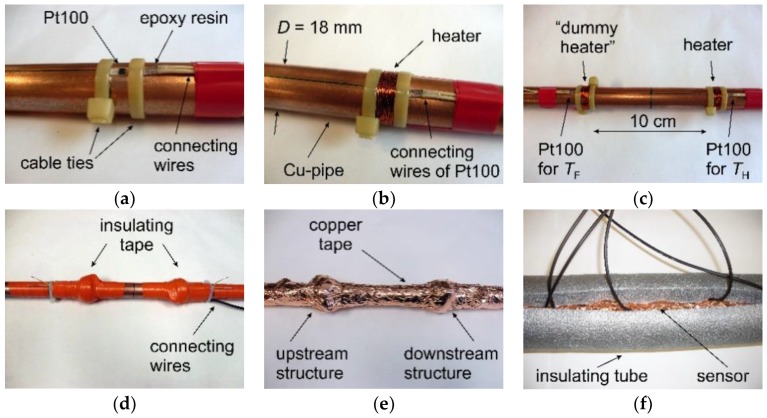
Construction steps of the first sensor prototype: (**a**) Pt100 element placed and tied on the copper pipe (inner diameter 16 mm, outer diameter 18 mm); (**b**) Copper winding around the Pt100 temperature sensor serves as a heater; (**c**) The same structure with a “dummy heater” was placed 10 cm upstream, in order to measure the fluid temperature *T*_F_; (**d**) Both structures were first insulated using electrical insulation tape; (**e**) Copper tape serves as a further insulation and shielding; (**f**) The last insulating layer consist of 2 cm thick polyethylene tube.

For the first sensor prototype, commercially available, miniaturized Pt100 elements were used (Figure 1b). One, measuring the heater temperature *T*_H_, was placed on the copper pipe surface and fixed with cable ties (Figure 2a). Subsequently, an 0.2 mm thin and 10 m long copper wire was wound around it forming the heater, which was supplied by a constant electrical current (Figure 2b). The electrical resistance of such a heater amounts to approximately 11 Ω at room temperature. This ensures that the electrical current needed to achieve the targeted heating power in the range of 5 to 10 W does not exceed the value of 1 A, for practical reasons. The second Pt100 element was positioned upstream of the heater at a distance of 10 cm to acquire an approximate value of the fluid temperature *T*_F_. If this distance is too low, the heater affects the temperature measurement. The required distance was estimated using a simple 2D-FEM model. Simulation results show that the surface temperature decreases rapidly with increasing distance upstream of the heater. Above a distance of about 10 cm, the influence of the heater on the measured fluid temperature can be neglected.

The same heater structure was also built around the upstream Pt100 element, however, without supplying it with electrical current (Figure 2c). Therefore, this “dummy heater” does not influence the temperature field, but significantly reduces transient response after sudden variations of fluid temperature, since both temperature sensors feature approximately the same thermal mass. For proper transduction, the complete assembly must be thoroughly insulated from the ambient. For that purpose, we first used a cotton gauze bandage followed by electrical insulation tape (Figure 2d). Besides the thermal insulation, the gauze bandage prevents the direct contact of the sticky plastic tape with the hot heater surface. A thin copper tape was applied as an electrical shielding layer (Figure 2e), which also helps to reduce effects of temperature gradients if they occur in the transducer’s ambient. And finally, the whole pipe with the sensor was thermally insulated from the ambient using polyethylene foam tube (Figure 2f). The insulating tube was subsequently tightened using cable ties.

## 3. Sensor Characterization

**Figure 3:**
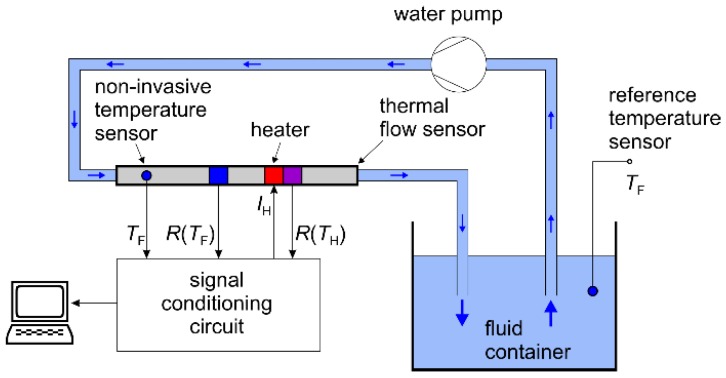
Schematic diagram of the measurement setup. *R*(*T*_H_) and *R*(*T*_F_) denote Pt100 elements located below and upstream of the heater, respectively. The heater is supplied with a constant electrical current *I*_H_. Water temperature *T*_F_ can be measured with a non-invasive sensor incorporated into the thermal flow sensor as well as with an invasive sensor used for the reference.

Figure 3 illustrates the simple measurement setup, which was used for all characterizations. It consists of an open fluid container, a water pump, and a closed pipe system incorporating the sensor prototype. In order to gain a reference for the fluid temperature *T*_F_, a Pt100 element was immersed in water. This temperature sensor has been used for measurements of transient responses after step-like changes of the fluid temperature (see Section 3.2, Figure 8a). An additional, non-invasive temperature sensor was integrated directly into the thermal flow sensors upstream of the “dummy heater” structure. It is needed to compensate the output signal drift due to variations of the fluid temperature, as it will be described later in Section 4. Varying the voltage of the water pump, step-like changes of the fluid flow velocity can be induced (see Section 3.2, Figure 8b). A signal conditioning circuit supplies the heater with the constant electrical current and collects all signals needed to derive the output value. It will be described in detail in the next section.

### 3.1. Steady-State Characterization

**Figure 4:**
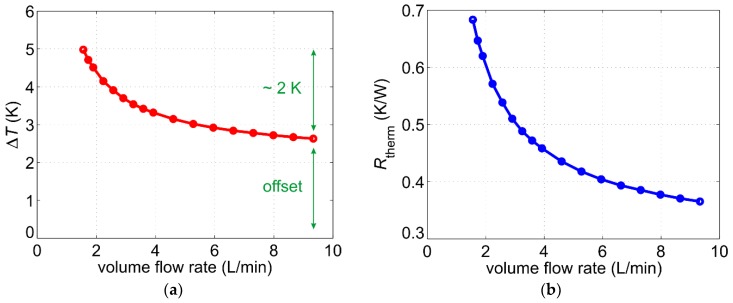
(**a**) Heater excess temperature as a function of the volume flow rate for a constant current supply of 780 mA resulting in a heating power of about 7.2 W; (**b**) Measured thermal resistance as a function of the volume flow rate.

In order to analyze the flow conversion, the heater was supplied with a constant electrical current of *I*_H_ = 780 mA. We used the *R*(*T*)-characteristic of the Pt100 devices to record the temperature difference between the heater and fluid temperature (Δ*T* = *T*_H_ − *T*_F_) as a function of flow velocity (Figure 4a). The sensor prototype was tested in a hydraulic system using water as a test fluid at room temperature of about 25 °C. The deployed water pump allows for volume flow rates $\dot{V}$ between 1.5 and 10 L/min resulting in mean flow velocities *v* in the pipe between approx. 0.1 and 0.8 m/s. The excess temperature Δ*T* depends on both the fluid velocity as well as the heating power *P*_H_. The latter lies for the applied heater current in the range of 7.2 W and features only a weak dependence on the flow velocity. This is due to resistance variations of the copper heater by varying flow velocities. The temperature coefficient of resistance for copper amounts to approximately $\alpha_{\mathrm{Cu}}\approx 0.004\;K^{-1}$ [15]. For increasing flow velocity, the heater temperature decreases (due to convective cooling), hence its resistance decreases too. As a result, the heating power decreases slightly with increasing flow velocity.

By dividing the measured excess temperature with the actual heating power for each velocity value we obtain the thermal resistance:

(1) $$R_{\mathrm{therm}}=\frac{\Delta T}{P_{H}}$$

that can be used to compare different sensors designs. It shows which excess temperature can be achieved for a heating power of 1 W. Especially, the slope of the *R*_therm_ characteristic is important since sensors with a steeper characteristic feature higher sensitivity. Figure 4b shows the measured thermal resistance as a function of the volume flow rate.

Two important issues arise from the excess temperature characteristic depicted in Figure 4a. First, the dynamic of the excess temperature (i.e., the signal variation over the desired measurement range) lies in a range of only 2 K. Platinum features a similar temperature coefficient of resistance as copper $\alpha_{\mathrm{Pt}}=\;\Delta R/\left(R\cdot \Delta T\right)\approx 0.4\%/K$ [16]. Temperature variation of 2 K results in relative resistance variation Δ*R*/*R* of less than 1% indicating that a high amplification accompanied by a thorough signal conditioning must be implemented. Secondly, the excess temperature characteristic features an offset, which arises from a high inherent temperature difference between the heater and the upstream Pt100 sensor. Higher dynamics of the excess temperature can be achieved by increasing the heating power. However, this will also increase the offset. Therefore, electronic amplifiers with variable offset correction must be used.

**Figure 5:**
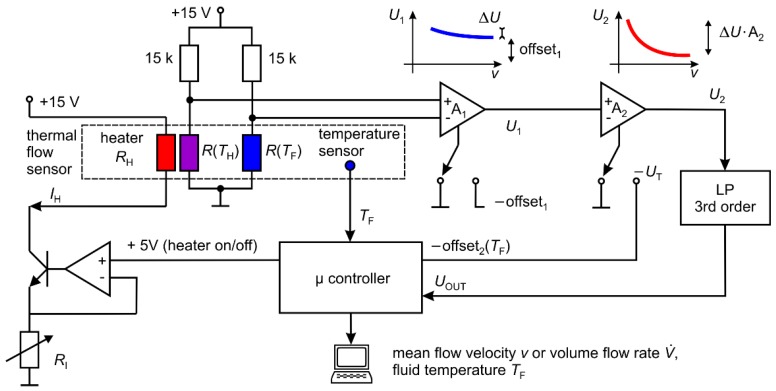
Schematic diagram of the signal conditioning circuit. *R*(*T*_H_) and *R*(*T*_F_) denote Pt100 elements located below and upstream of the heater, respectively. Adjusting the offset of the second amplifier, the temperature dependence of the output signal can be taken into account. Before being sampled by a microcontroller, the output signal is filtered by a 3rd order low pass filter with a cutoff frequency of 2 Hz.

The schematic diagram of a signal conditioning circuit developed to meet the mentioned demands is depicted in Figure 5. It consists of a Wheatstone bridge and two amplifiers with optional offset correction. The electrical current through the Pt100 elements amounts to approximately 1 mA ensuring that self-heating effects are minimized. When the offset correction of the first instrumentation amplifier is turned off, its output signal *U*_1_ correlates to the heater excess temperature depicted in Figure 4a. This signal contains an offset voltage (denoted as offset_1_ in Figure 5) that arise from a high inherent temperature difference between the heater and the upstream Pt100 sensor. It must be compensated to enable further amplification of the flow-dependent variations. The offset corrected signal is normalized to the desired output range by the second amplifier in order to obtain the best readout sensitivity.

Additionally, the output signal *U*_2_ depends on the fluid temperature *T*_F_. The exact temperature dependence is a function of several factors such as fluid parameters, pipe and heater material, or overall amplification (A_1_∙A_2_) and must be determined experimentally (see Section 4). It can be considered by measuring the fluid temperature *T*_F_ with a separate, non-invasive temperature sensor and applying an appropriate offset at the second amplifier (denoted as offset_2_ or *U*_T_ in Figure 5). In order to reduce noise influences, the offset corrected output signal was finally filtered by a 3rd order low pass filter.

A low-cost microcontroller (Arduino Uno) was used to sample the output signal *U*_OUT_ and to display current measurement results. It also monitors the water temperature *T*_F_ and applies the corresponding temperature correction voltage *U*_T_ on the second amplifier. Finally, it switches the heating on and off.

The schematic diagram of the constant current source can be seen in the left part of Figure 5. The heater current *I*_H_ can be adjusted by the resistor *R*_I_. For the following characterizations of the first sensors prototype, the heater current amounts to 730 mA resulting in a heating power in the range between 6.3 W and 6.4 W. The overall amplification and the offset_1_ were chosen such that the sensor output signal fits between 0 V and 5 V in the flow range of interest. Hence, the output can be immediately sampled by the deployed microcontroller.

**Figure 6:**
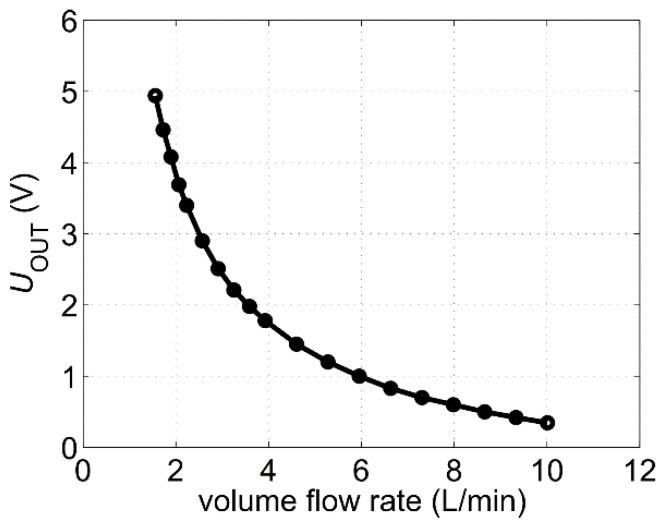
Output characteristic of the first sensor prototype. The following parameters have been chosen in the measurement: A_1_ = 1000, A_2_ = 6.3, *I*_H_ = 730 mA, water temperature was around the reference value of 25 °C, offset_1_ ≈ 730 mV while the offset of the second amplifier was switched off.

The obtained conversion characteristic is depicted in Figure 6. The flow conversion is most effective for low fluid velocities. For higher flow velocities, the signal saturates and the sensitivity decreases. As this transduction function was measured at the reference fluid temperature of 25 °C, it is used as a reference characteristic for the microcontroller. Applying the inverse function of the conversion characteristic depicted in Figure 6, the microcontroller calculates the volume flow rate or alternatively the mean flow velocity inside the pipe.

### 3.2. Transient Characterization

**Figure 7:**
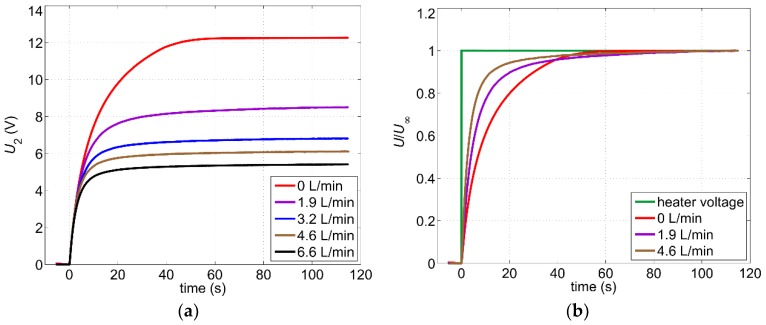
(**a**) Transients of the output signal for different volume flow rates in response to switching on the heater power; (**b**) Corresponding normalized transients. *U*_∞_ denotes the steady-state value reached after approx. 2 min. Measurements were conducted with a digital oscilloscope using averaging mode to suppress noise effects.

As copper is a good heat conductor and common fluids exhibit high specific heat values, a relatively high heating power must be applied in order to obtain the required excess temperature for a reasonable output signal. For applications where continuous measurement is not required, intermittent operation can help to reduce the average heating power consumption. In this case, the heating is switched on for a period required to take a single velocity value, while for the rest of the time the sensor returns into a zero-power mode. However, after the heating is switched on, the thermal transient must be awaited to reach the steady-state before the measurement can start. In order to determine this adjustment period, transients of the output signal at different flow velocities have been recorded. The results are illustrated in Figure 7a.

For this measurement, the offsets of both amplifiers have been switched off and the non-filtered output signal *U*_2_ has been acquired with a digital oscilloscope. The steady-state values after about 120 s correspond to the measured values given in Figure 6 (except for an offset of about 4.67 V = offset_1_·A_2_). The rise time τ_r_ (defined as a time required for the output signal to rise from 10% to 90% of its steady value) depends on the flow velocity. This can be best illustrated if transient responses are related to the steady-state values as shown in Figure 7b. The longest rise time of about 30 s has been reached for zero flow. For volume flow rates between 1.5 and 10 L/min, the rise time decreases to 10–20 s.

The dependence of the rise time on the flow velocity can be explained by the lumped-heat-capacity method. As the resistance to heat transfer by conduction through the copper layer (heater and pipe) is small compared with the convection resistance at the inner pipe wall, this simplified analysis can be applied [17]. Here, the thermal system can be represented by an analog electrical system consisting of an RC circuit. The time constant *τ* describing charging and discharging of the capacitor (switching the heater on and off) can be written as:

(2) $$\tau =R_{\mathrm{th}}\cdot C_{\mathrm{th}},\;R_{\mathrm{th}}=\frac{1}{hA},\;C_{\mathrm{th}}=\rho cV,$$

where *R*_th_ and *C*_th_ denote thermal resistance and capacitance, respectively. *V* is the volume of the heater and the pipe section around the heater, *A* is the inner surface of the corresponding pipe section, whereas *h*, *ρ*, and *c* denote the convection heat-transfer coefficient, fluid density, and specific heat, respectively. For increasing volume flow, the heat-transfer coefficient *h* increases too, resulting in shorter rise times. The measurement results depicted in Figure 7 reveal that for intermittent operation a heating period of at least two minutes is mandatory before the recording of the flow velocity can be started.

**Figure 8:**
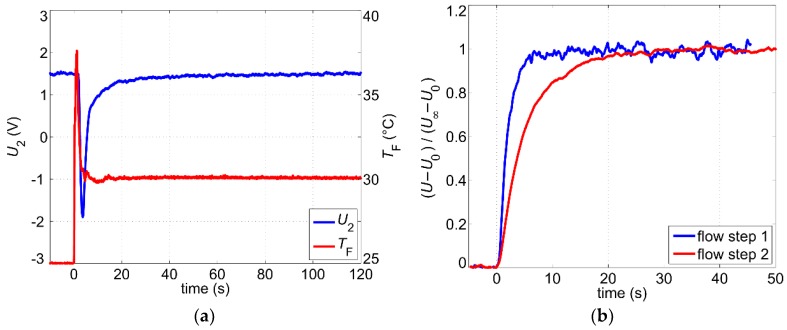
(**a**) Transient response of the output signal after a step-like change of the fluid temperature at a constant volume flow rate of 4.6 L/min; (**b**) Transient response of the output signal after step-like changes of the volume flow rate (flow step 1: from 8.7 L/min to 4.9 L/min, flow step 2: from 4.6 L/min to 1.9 L/min). *U*_0_ and *U*_∞_ denote the initial and the final value, respectively.

Even after a steady-state has been reached, variations of the fluid temperature still influence the output signal. In order to quantify this effect, we studied the transient response of the output signal to abrupt changes of the fluid temperature. As described in Section 3, the measurement setup consists of an open 5 L water reservoir, kept at room temperature. Step-like changes of the water temperature were induced by pouring hot water into the reservoir. Figure 8a shows the transient response of the non-filtered output signal *U*_2_ after the water temperature was abruptly changed from 25 °C to 30 °C. The water temperature was measured using the reference Pt100 sensor immersed into the water container (see Figure 3). During the measurement, the volume flow rate was kept constant at 4.6 L/min.

The warmed-up water reaches at first the upstream temperature sensor causing the excess temperature Δ*T* = *T*_H_ − *T*_F_ to decrease. Hence, the output signal drops until the warm fluid arrives at the downstream sensor. Subsequently, the output signal increases steadily, reaching 95% of the steady-state value in about 30 s. After about 2 to 3 min the transient response can be assumed as concluded.

The response to the abrupt changes of the flow velocity is another important aspect of the transient sensor characterization. The step-like changes of the volume flow rate were induced by a sudden variation of the supply voltage on the water pump. The delay arising from the dynamic response of the water pump is much shorter than the signal response and can be neglected. Changes of the flow velocity influence the thicknesses of both the hydrodynamic and the thermal boundary layer, which in turn affects the excess temperature. After step-like changes of the flow velocity, the boundary layers are reshaped to adapt to the new hydrodynamic conditions. Therefore, the response time depends on the initial and the new value of the flow velocity. Normalized transient responses for two different flow steps are shown in Figure 8b. The lower the flow velocity, the thicker the boundary layers, and hence, the rise time increases. For the first flow step, where the volume flow changes from a high volume flow of 8.7 L/min to a medium value of 4.9 L/min, the rise time (10–90%) amounts to only τ_r_ ≈ 3.6 s. In the second flow step, the volume flow changes from the medium volume flow of 4.6 L/min to a very low value of 1.9 L/min and the rise time increases to τ_r_ ≈ 12.3 s. In order to suppress noise effects, a moving average procedure with a window size of 32 ms was applied to the measured signals depicted in Figure 8. This low window size does not affect the measurement of the rise time but enables clear diagrams.

The observed response times are decisive for the adjustment of the cutoff frequency of the electrical low pass filter (see Figure 5). Moreover, the sensor transduction features also a low pass characteristic regarding variations of the flow velocity. If we approximate the thermal system as a low pass filter of first order, then the cutoff frequency reads [18]:

(3) $$f_{c}=\frac{\ln9}{2\pi \cdot \tau_{r}}\approx \frac{0.35}{\tau_{r}},$$

where τ_r_ denotes the rise time (10–90%). The fastest rise times in the order of a few seconds have been measured at high flow velocities (Figure 8b). Inserting those values in Equation (3) yields cutoff frequencies in the range of hundreds of mHz. Hence, the electrical low pass filter with a cutoff frequency in the order of 1 Hz (as implemented for this study) is not influencing the dynamics of the output signal but helps to reduce the noise influence and thus to improve the signal-to-noise ratio.

### 3.3. Noise Analysis

**Figure 9:**
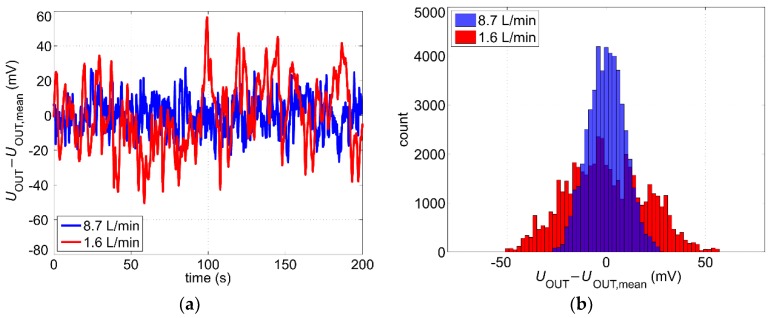
Oscillograms (**a**) and the corresponding histograms (**b**) of the zero-mean output voltage *U*_OUT_ − *U*_OUT,mean_ for two different volume flow rates.

As can be seen from Figure 8b, the steady-state output signal contains significant low-frequency noise components. Obviously, these fluctuations depend on the flow velocity and limit the ultimate accuracy of the measurement. In order to study noise-related effects, the steady-state output signal after the low pass filter *U*_OUT_ was sampled by a digital oscilloscope at different volume flow rates. We recorded periods of 200 s at a sampling frequency of 250 Hz. Figure 9a shows oscillograms in case of a high and a low volume flow rate. Histograms with corresponding 50000 samples suggest a Gaussian distribution of the noise (Figure 9b).

**Figure 10:**
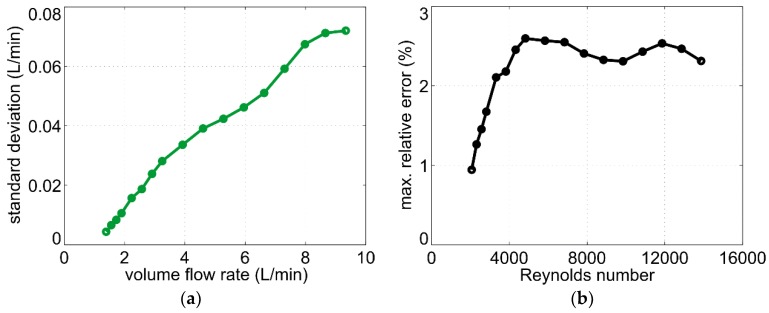
(**a**) Standard deviation $\sigma_{\dot{V}}=\sigma_{U}/S$ as a function of the volume flow rate; (**b**) Estimation of the maximum relative error (Equation (6)) as a function of the Reynolds number.

Figure 9b suggests furthermore that the standard deviation of the output voltage decreases with increasing volume flow. To estimate the actual standard deviation for certain flow velocity, we first performed a series of twelve data recordings (as shown exemplarily in Figure 9a) determining a standard deviation for each of those 200 s long data sets. Then, using these results we calculated a mean value for the standard deviation of the output voltage $\sigma_{U}$. Finally, in order to obtain the standard deviation of the volume flow rate (as the actual output quantity), $\sigma_{U}$ must be scaled with the reciprocal of sensitivity:

(4) $$S=\left|\frac{dU_{\mathrm{OUT}}}{d\dot{V}}\right|,$$

where *S* reflects the slope of the output characteristic depicted in Figure 6. The results are shown in Figure 10a. The standard deviation of the volume flow rate $\sigma_{\dot{V}}=\sigma_{U}/S$ increases with an increasing flow velocity, which can be attributed to the turbulent flow regime inside the pipe.

The Reynolds number for a pipe flow can be defined as [17]:

(5) Re=ρvDμ,

where *ρ* and μ denote the density and the dynamic viscosity of the fluid, respectively, *D* is the pipe diameter and *v* the mean flow velocity in the pipe. For water as fluid at room temperature, a pipe diameter of *D* = 16 mm, and volume flow rates from 1.5 L/min to 10 L/min, the Reynolds number ranges between 2000 and 16000. For hot water, the viscosity decreases and the Reynolds number becomes even higher. Therefore, a turbulent velocity field can be assumed inside the pipe for all values of the measured volume flow rate. For increasing flow rate, the eddy velocity increases too, resulting in a higher standard deviation $\sigma_{\dot{V}}$ as shown in Figure 10a.

About 99.7% of values drawn from a normal distribution lie within three standard deviations around the mean. Assuming normal distribution for the measured volume flow rate (see Figure 9b), we can, therefore, estimate the maximum relative error as:

(6) $$|E_{r,\max}|=\frac{3\sigma_{\dot{V}}}{\dot{V}}\cdot 100\%,$$

where $\sigma_{\dot{V}}$ is the measured standard deviation for a certain volume flow rate $\dot{V}$. The maximum relative error as a function of Reynolds number is shown in Figure 10b. In the range 2000 < Re < 4000 (transition from laminar to turbulent flow), the relative error increases with rising Reynolds number. After the flow becomes fully turbulent (Re > 4000 [17]), the standard deviation increases proportionally to the volume flow rate and, hence, the relative error remains approximately constant over the higher range of the Reynolds number. Nevertheless, the maximum relative error lies below 3% for all investigated flow rates up to 10 L/min.

**Figure 11:**
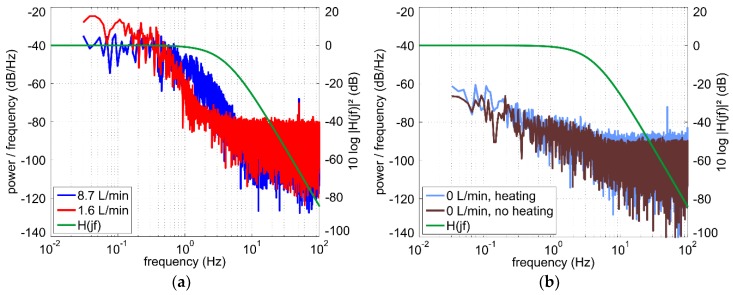
(**a**) Power spectral density estimates (periodograms) of the output voltage *U*_OUT_ for two different volume flow rates. H(jf) denotes the transfer function of the applied third-order low-pass filter; (**b**) Periodogram of the output voltage for zero flow with and without the heating.

A closer look at the oscillograms illustrated in Figure 9a reveals that higher flow velocity correlates with increased high-frequency fluctuations of the output voltage. Power spectral density of the noise can be obtained by means of fast Fourier transform (FFT) of the recorded signal sequences. Figure 11a shows periodograms [19] calculated using the data sets depicted in Figure 9a. For better comparability, the gain-magnitude frequency response of the applied third-order low-pass filter is shown too. This filter consists of three passive first-order low pass filters, each of them featuring the same cutoff frequency of 4 Hz. As these filters are separated by buffer amplifiers, the overall cutoff frequency reduces to about $f_{c}=4\mathrm{Hz}\cdot \sqrt{\sqrt[{3}]{2}-1}$ ≈ 2 Hz [20]. The same measurements were also performed with one first-order low pass filter as well as with just a broad built-in filter from the oscilloscope. Spectral comparison shows that the low pass filters effectively reduce the base level of the frequency independent broadband noise in the right part of Figure 11a.

The $1/f^{\alpha }$-noise in the left part of the diagram depends on the flow velocity. Besides the disturbances in the flow, thermal coupling between the heater and Pt100 elements as well as the sensor electronic can be possible noise sources. To estimate their influence we recorded output voltage at zero flow with and without the heating. At zero flow velocity with heating, the output voltage drifts slowly due to natural convection. Removing this drift from the measured output voltage and performing a FFT, we obtain the periodogram in Figure 11b (light-blue characteristic). Moreover, the periodogram at zero flow without heating is depicted too. This noise stems merely from the sensor electronic (brown characteristic). A comparison of Figure 11a and 11b confirms that turbulent flow in the pipe is the main source of output noise. As it can be seen in Figure 11a, the $1/f^{\alpha }$-noise components shift to higher frequencies for increasing flow velocity. However, these fluctuations are not a consequence of a malfunction of the transducer as they are tightly related to the stochastic nature of the turbulent flow velocity field.

Influence of the $1/f^{\alpha }$-noise cannot be completely suppressed by simply decreasing the cutoff frequency and increasing the order of the low pass filter. A more elegant way to reduce its influence is to apply averaging of the sampled output signal. For example, a moving average procedure seems to be a good solution to suppress high-frequency contributions as it does not reduce the rate of the output signal. The appropriate window size can be estimated through transient characterization. Transient responses after step-like changes of the fluid temperature or volume flow rate can last more than 30 s (see Figure 8). Therefore, in order to reduce the effects of the $1/f^{\alpha }$-noise, we used signal averaging over 30 s for long-term measurements presented in the next section.

## 4. Compensation of Temperature Dependence and Long-Term Measurements

The output voltage *U*_OUT_ is a function of the heater excess temperature, which in turn depends on the heating power and the thermal resistance $\Delta T=P_{H}\cdot R_{\mathrm{therm}}=I_{H}^{2}\cdot R_{H}\cdot R_{\mathrm{therm}}$ (see Equation (1)). As mentioned earlier in Section 3.1, the temperature coefficient of resistance for copper amounts to $\alpha_{\mathrm{Cu}}\approx 0.004\;K^{-1}$. The electrical resistance of the copper heater coil *R*_H_ depends therefore on the absolute heater temperature $T_{H}=\Delta T+T_{F}\;$and, hence, on the fluid temperature *T*_F_. For constant current supply *I*_H_, the heating power $P_{H}=I_{H}^{2}\cdot R_{H}$ and, thus, Δ*T* depends also on the water temperature inside the pipe. Consequently, in this operating mode, the fluid temperature must be monitored during the measurement in order to correct the output voltage and obtain the proper flow rate.

There are two other operating modes commonly used to reduce the dependence of the output signal on the fluid temperature. The first one uses a closed-loop control system in order to keep the heating power constant and, hence, independent on the fluid temperature. In the second alternative mode, the controller keeps the excess temperature constant. In this case, the heating power *P*_H_ = Δ*T*/*R*_therm_ required to maintain the desired value of Δ*T* serves as the output quantity.

In the course of this work, both additional operating modes were realized. However, no significant reduction of the temperature dependence of the output signal could be observed. This is mainly due to the temperature dependence of the thermal resistance (see Equation (1)). *R*_therm_ characterizes the cooling of the heater and depends not only on fluid velocity but also on material parameters of the fluid and the pipe wall. Those parameters such as mass density or thermal conductivity are temperature dependent and, therefore, variations of the fluid temperature influences the value of the thermal resistance. Due to the high overall amplification (A_1_·A_2_ = 6300), even a small variation of *R*_therm_ has a large impact on the output signal. Consequently, the output signal depends on the fluid temperature even if the heating power *P*_H_ or excess temperature Δ*T* are kept constant. A detailed description of temperature dependence for each operating modes goes beyond the scope of this work. Here, we point out that independent of the applied operating mode (constant current, constant heating power, or constant excess temperature mode), a compensation of the temperature dependence must be applied for proper detection of the flow rate.

**Figure 12:**
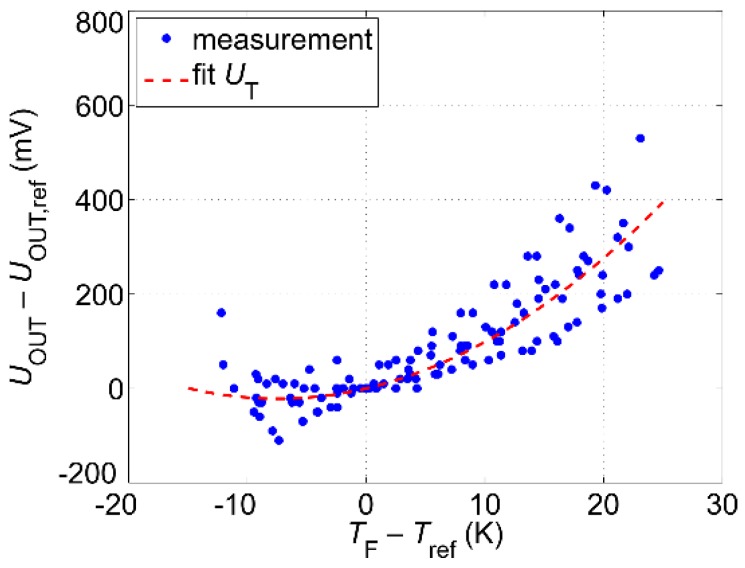
Difference between output voltage *U*_OUT_ (measured at fluid temperature *T*_F_) and the reference value *U*_OUT,ref_ (measured at reference fluid temperature *T*_ref_ = 25 °C) as a function of temperature difference *T*_F_ − *T*_ref_. In the measurement, *T*_F_ was varied from 10 °C to 50 °C and then back to 10 °C for three constant volume flow rates (1.9 L/min, 4.6 L/min, and 8.7 L/min).

The constant current mode is the simplest operating mode as it does not require a closed loop control system. Therefore, we focused on this operating mode. In order to derive the correction function, the fluid temperature was varied between 10 °C and 50 °C for three representative flow rates at the beginning, in the middle, and at the end of the flow range. Figure 12 shows the deviation of the measured output voltage *U*_OUT_(*T*_F_) from the reference value *U*_OUT,ref_ = *U*_OUT_(*T*_ref_) as a function of the temperature difference *T*_F_ − *T*_ref_. The room temperature was kept constant during all measurements and serves as a reference value for the temperature (*T*_ref_ = 25 °C). 

The voltage difference *U*_OUT_ − *U*_OUT,ref_ depends not only on the fluid temperature *T*_F_ but also on the flow rate and the way how *T*_F_ has been changed (i.e., if the fluid temperature during the measurement increases or decreases). As a consequence, the set of measurement values features significant spread. Therefore, a least-square fit (red dashed line in Figure 12) serves as a correction function *U*_T_. In operation, the microcontroller monitors the fluid temperature *T*_F_ with an additional non-invasive temperature sensor (see Figure 5) and calculates the offset of the second amplifier −*U*_T_(*T*_F_). In this case, the output voltage sampled by the microcontroller reads *U*_OUT,ref_ = *U*_OUT_ − *U*_T_. Finally, by applying the inverse function of the reference characteristic depicted in Figure 6 volume flow or mean flow velocity can be calculated and displayed as output.

This concept was tested by long-term measurements at constant flow velocity but variable fluid temperature. During a period of one day, we first increased the fluid temperature up to about 50 °C and then left the hydraulic system to cool down slowly. Afterwards, the water temperature was decreased down to approximately 10 °C followed by a warm-up phase back to room temperature. The used water pump suits for long-term operation only for volume flow rates below 5 L/min. Therefore, we performed two different long-term measurements for 1.9 L/min and 4.6 L/min.

For the first measurement series, the heating was permanently switched on and the flow rate was set to 1.9 L/min. During a period of 30 s, the microcontroller samples one hundred values of the output voltage followed by an idle period of the next 30 s without data sampling. A mean value from the recorded hundred samples is then used to calculate the actual flow rate. Hence, the measurement rate in this operating mode amounts to 60 values per hour. The relative error can be calculated from:

(7) $$E_{r}=\frac{{\dot{V}}_{\mathrm{meas}}-{\dot{V}}_{\mathrm{set}}}{{\dot{V}}_{\mathrm{set}}}\cdot 100\%,$$

where ${\dot{V}}_{\mathrm{meas}}$ and ${\dot{V}}_{\mathrm{set}}$ denote the measured and the reference value of the volume flow rate, respectively.

**Figure 13:**
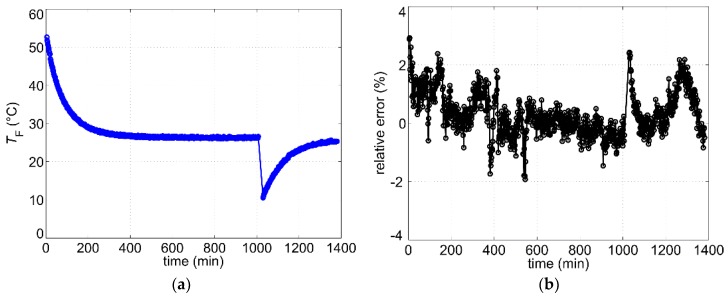
(**a**) Measured fluid temperature during long-term measurement with continues heating and constant flow rate of ${\dot{V}}_{\mathrm{set}}=1.9$ L/min; (**b**) Corresponding relative error (Equation (7)).

Figure 13a illustrates the variation of the fluid temperature over a period of about 24 h. The corresponding relative error of the measured volume flow rate is depicted in Figure 13b. The maximum overall relative error amounts to about 3%. During the period with constant fluid temperature (between 600 min and 1000 min) it decreases to approximately ± 1%.

**Figure 14:**
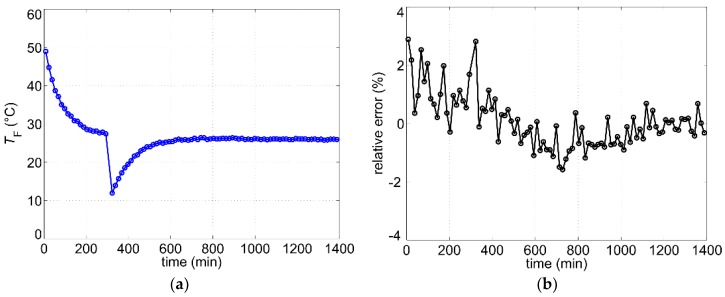
(**a**) Measured fluid temperature during long-term measurements with intermittent heating and constant flow rate of ${\dot{V}}_{\mathrm{set}}=4.6$ L/min; (**b**) Corresponding relative error (Equation (7)).

In the second measurement series, the heating was alternatingly switched on and off for 6.5 min whereas the flow rate was set to 4.6 L/min. Due to the duty cycle of 50%, the average heating power was reduced by a factor of 2. During the last 30 s of the heating period, the microcontroller records 100 samples and calculates the flow rate using the mean. The average measurement rate in this operating mode amounts to less than five values per hour. The variation of the fluid temperature over a period of about 24 h for this operating mode can be seen in Figure 14a. Again, the measured relative error (Figure 14b) does not exceed the value of 3%, whereas the relative error at a constant fluid temperature (between 800 min and 1400 min) is in the range of about ± 1%.

The two presented long-term measurement series prove the feasibility of the automatic compensation of temperature dependence. Moreover, no significant drift effects of the output signal were observed during the measurements.

## 5. Second Sensor Prototype

The sensor prototype presented in the preceding sections was built to demonstrate the feasibility of non-invasive measurements of the flow rate utilizing thermal transduction principles. It features a very simple and cost-effective approach but its main drawback is that the heater coil cannot be retrofitted easily in an existing hydraulic system. Therefore, we considered a design improvement consisting of Pt100 elements and flexible PCB heaters with integrated self-adhesive foil for easier mounting.

**Figure 15:**
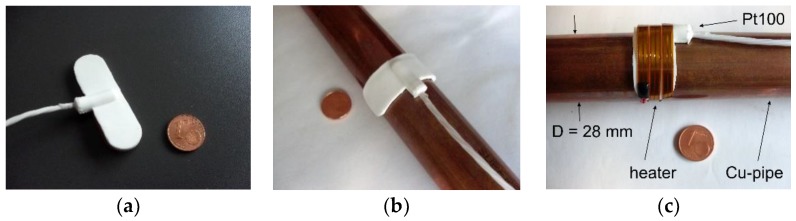
(**a**) Pt100 elements used for the second sensor prototype; (**b**) Pt100 element affixed on the surface of a copper pipe (inner diameter 26 mm, outer diameter 28 mm); (**c**) Self-adhesive heater stripe wound around the Pt100 temperature sensor.

Figure 15a shows a commercially available Pt100 temperature sensor that was used for the second sensor prototype. It is incorporated in a flexible silicone rubber carrier with dimensions of 5 cm × 1.5 cm and a thickness of about 1 mm. The platinum layer is bonded onto a self-adhesive aluminum foil strip at the bottom of the sensor in order to improve the sensor response to nearby temperature variations. The sensor could be damaged if bent around thin pipes as used for the first sensor prototype. Therefore, the second prototype was attached to a copper pipe with a larger diameter of 28 mm (Figure 15b). Around the Pt100 element, a 4 mm wide and 253 mm long self-adhesive polyimide heater was wound covering completely the outer surface of the temperature sensor (Figure 15c). All subsequent mounting steps are the same as with the first sensor prototype (see Figure 2c–f).

Due to the thick silicon rubber layer as well as the self-adhesive aluminum foil strip, the Pt100 elements feature a relatively high thermal mass and a moderate thermal conductance in the radial direction. Moreover, they cover more than half of the pipe circumference. The thermal coupling between the heater and the pipe transporting the fluid is therefore reduced.

**Figure 16:**
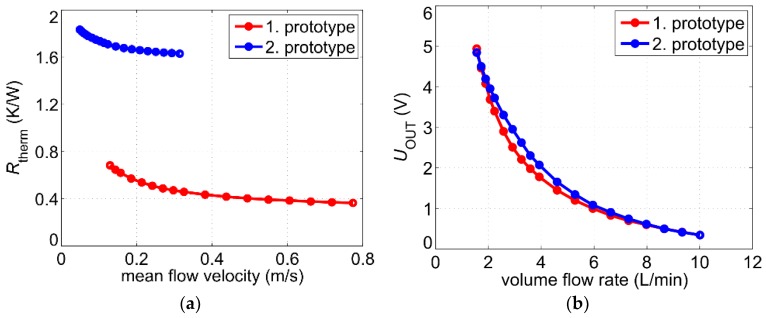
(**a**) Thermal resistance of both sensor prototypes as a function of the mean flow velocity; (**b**) Comparison of the output characteristics. The following parameters have been chosen for the second prototype: A_1_ = 1000, A_2_ = 12.5, *I*_H_ = 780 mA, *T*_F_ ≈ 25 °C, offset_1_ ≈ 2840 mV while the offset of the second amplifier was switched off. In contrast, the parameters of the first prototype are summarized in Figure 6.

The self-adhesive heater strip features an electrical resistance of about 8 Ω. The characterization of flow conversion was performed using a constant current supply of *I*_H_ = 780 mA resulting in a heating power of about 4.9 W. This is somewhat less than the heating power applied to the first sensor prototype. Hence, in order to compare the transduction efficiency of sensor prototypes, the characteristic of the excess temperature is not suitable. Instead, the characteristic of the thermal resistance must be used as it does not depend on the applied heating power. However, the pipe diameters of the two sensor prototypes are not identical. As a consequence, the mean flow velocity inside the pipes differs even for the equal volume flow rate adjusted by the water pump. As the convective cooling depends directly on the mean flow velocity *v*, we used this variable to compare the thermal resistance characteristics (Figure 16a). Because of the design differences described above, the excess temperature of the second prototype is much higher for the same heating power. However, the high excess temperature influences only the offset voltage of the first amplifier (denoted as offset_1_ in Figure 5). A more important parameter is the slope of the characteristics. The difference between thermal resistance at the highest and lowest flow velocity *R*_therm,max_ − *R*_therm,min_ is by a factor of 2 lower for the second sensor prototype. This is mainly due to weaker thermal coupling and can be considered by increasing the gain of the second amplifier (denoted as A_2_ in Figure 5). 

By adjusting the parameters offset_1_ and A_2_ of the sensor electronic circuit, it can be ensured that the output characteristic fits between 0 V and 5 V in the volume flow range provided by the water pump. A comparison between the output characteristics regarding the volume flow rate as an input variable shows only minor differences (Figure 16b). Consequently, the same electronic circuit and controller can be used for the second sensor prototype provided that above-mentioned parameters, as well as the inverse function of the output characteristic, are changed accordingly. Moreover, the correction function *U*_T_(*T*_F_) must be derived anew as it depends on the material of the heater.

**Figure 17:**
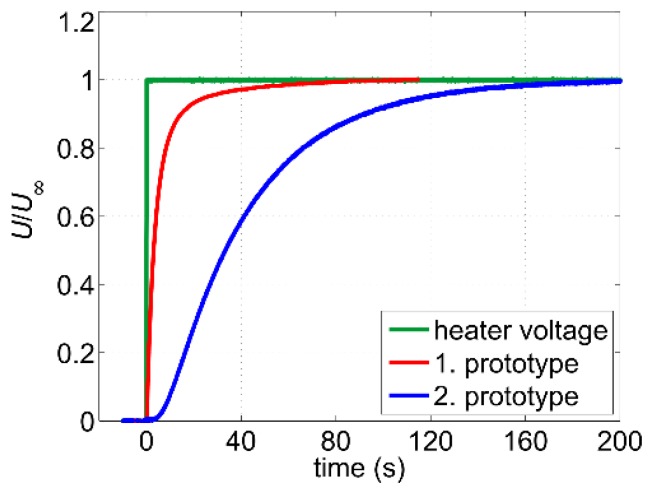
Comparison of the normalized transients of the output signal in response to switching on the heater power. *U*_∞_ denotes the steady-state value. For both sensor prototypes, the same constant mean flow velocity of about 0.27 m/s was adjusted.

The increased thermal mass of the self-adhesive Pt100 elements has a negative impact on the sensor’s dynamic behavior. Figure 17 shows transient responses of the output voltage at a constant flow after the heating has been switched on. The volume flow rate during the measurements was adjusted to obtain the same mean flow velocity in the pipe for both sensor prototypes. Whereas the first prototype features no delay and a 10–90% rise time of approximately 14 s, the second prototype is much slower with a dead time of about 3 s and a rise time of approximately 80 s. Therefore, if the second sensor prototype is going to be operated in the intermittent mode, the heating must be switched on for at least four minutes before the recording of the flow velocity can be started. This is twice as long as it was the case with the first sensor prototype (see Figure 7b).

**Figure 18:**
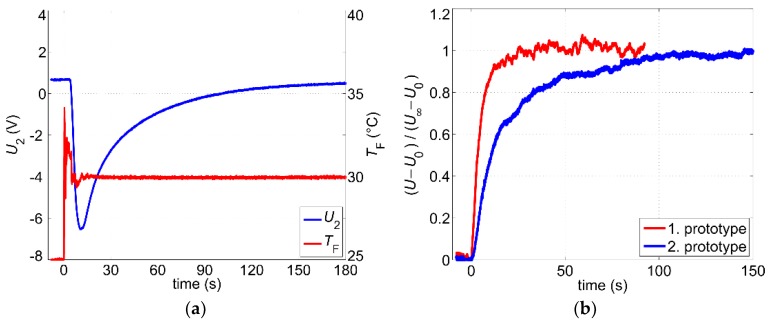
(**a**) Transient response of the output signal of the second sensor prototype after a step-like change of the fluid temperature at a constant volume flow rate of 9.3 L/min; (**b**) Comparison of transient responses after step-like changes of the mean flow velocity (flow step from 0.27 m/s to 0.17 m/s). *U*_0_ and *U*_∞_ denote the initial and the final value, respectively.

Moreover, the design of the self-adhesive elements has a negative influence on the signal dynamic also for continuous heating after thermal steady-state has been reached. Figure 18a illustrates the transient response of the second prototype to an abrupt change of the water temperature at a constant flow rate of about 9.3 L/min. The course of the output signal is similar to the one depicted in Figure 8a. However, the output signal of the second prototype needs more than 170 s to reach 95% of the steady-state value, which is about six times longer than the response time of the first sensor prototype. Although the mean flow velocity during the measurements was not the same (about 0.38 m/s for the first prototype and 0.29 m/s for the second prototype), the comparison with Figure 8a confirms the trend of prolonged response times for the second prototype.

A comparison of normalized transient responses after step-like changes of the flow velocity is shown in Figure 18b. For both measurements, the mean flow velocity decreases abruptly from 0.27 m/s to about 0.17 m/s. Again, the prototype with smaller Pt100 elements features a much shorter reaction time. The rise time (10–90%) of the first prototype amounts to only τ_r_ ≈ 11 s while for the second prototype it increases by a factor of 5 to τ_r_ ≈ 56 s.

However, the use of large Pt100 elements introduces also some benefits. As Figure 18b suggests, the steady-state output signal of the second prototype contains significantly fewer noise components compared to its predecessor. The extension of the self-adhesive Pt100 elements causes a spatial averaging of thermal field variations resulting in a noise reduction. Moreover, due to higher thermal mass compared to the temperature sensors of the first prototype, the bulky Pt100 elements react more slowly (Figure 18b), which indicates a decrease in bandwidth and hence a reduction of noise effects.

**Figure 19:**
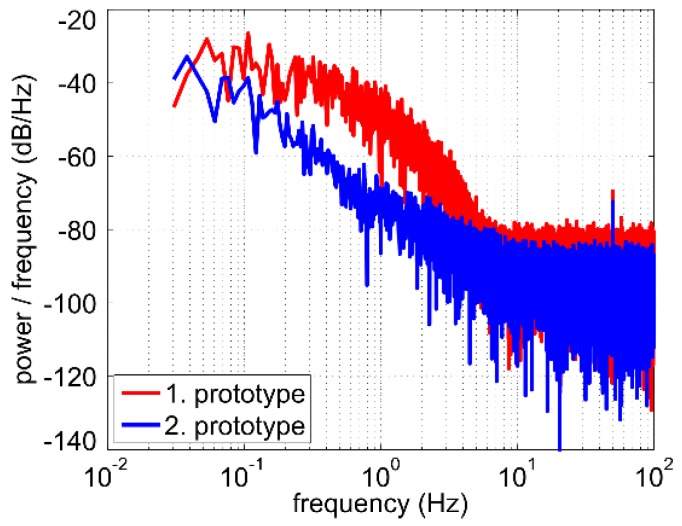
Power spectral density estimates (periodograms) of the output voltage *U*_OUT_ at a constant Reynolds number of Re ≈ 8000 for both sensor.

Figure 19 depicts a comparison of the periodograms of the output voltage *U*_OUT_ of both prototypes. The volume flow rate during signal sampling was adjusted to obtain approximately the same Reynolds number in both cases. Depending on the frequency range, the $1/f^{\alpha }$-noise of the output voltage of the second prototype is reduced up to 20 dB. 

**Figure 20:**
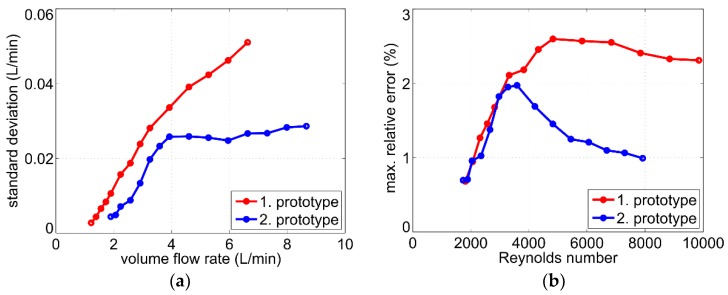
(**a**) Comparison of the standard deviation $\sigma_{\dot{V}}=\sigma_{U}/S$ as a function of the volume flow rate; (**b**) Comparison of the maximum relative error (estimation according to Equation (6)) as a function of the Reynolds number.

This has also an impact on the sensor accuracy. Figure 20a illustrates the estimated standard deviation $\sigma_{\dot{V}}=\sigma_{U}/S$ as a function of the volume flow rate for both sensor prototypes in comparison. The standard deviation of the second prototype is thereby lower for all volume flow rates. One reason is the reduced noise influence as described above. Another reason is the smaller pipe diameter of the second prototype resulting in lower mean flow velocities in the pipe at the same volume flow rates and, consequently, in weaker turbulent disruptions.

For better comparison, the dependence on the Reynolds number should be used. This was done in Figure 20b, which shows the maximum relative error of both sensor prototypes estimated according to Equation (6). For low flow velocities (Re < 4000), the standard deviation of the volume flow rate $\sigma_{\dot{V}}=\sigma_{U}/S$ for both sensor prototypes increases disproportionately with the flow velocity. Hence, the relative error of both prototypes increases with rising Reynolds numbers. For fully developed turbulent flow, the standard deviation $\sigma_{\dot{V}}$ of the first prototype increases almost proportionally to the flow velocity (red characteristic in Figure 20a for about $\dot{V}>3$ L/min) resulting in a nearly constant relative error over the Reynolds number. 

On the contrary, due to the described averaging effects, the standard deviation $\sigma_{\dot{V}}$ of the second prototype increases only slightly at higher flow velocities (blue characteristic in Figure 20a for about $\dot{V}>4$ L/min). Therefore, for Re > 4000 its relative error decreases and ranges between 1% and 2% (Figure 20b).

## 6. Discussion and Conclusions

In this paper, we presented a feasibility study on non-invasive flow rate measurements in metal pipes utilizing low-cost thermal flow transducers. We demonstrated that thermal flow sensors based on a modified hot-film transduction principle can be operated successfully at heating powers far less than 10 W. Further reduction can be achieved by optimizing the sensor electronics, which will be a topic of future research. Moreover, applying the intermittent operation helps to reduce the *average* power consumption.

The first investigated sensor prototype consists of two miniaturized Pt100 elements attached to the copper tubing with a copper wire wound around them. One coil was operated as a heater supplied by a constant current whereas the other one was not in use. However, this “dummy” heater is of significant importance for reduction of the transient time after step-like changes of fluid temperature.

In order to address the issue of long response times, special attention was paid to a thorough transient characterization. The shortest response times in the order of 10 s (or even less, depending on the initial and steady-state value of flow steps) were achieved with the first sensor prototype featuring small Pt100 elements. Due to the coiled heater, the first prototype was difficult to mount and served only as a proof of principle.

The second prototype utilizes self-adhesive components, which can easily be mounted within an HVAC system. Moreover, the spatial averaging of thermal field variations due to extended Pt100 elements as well as the decrease in bandwidth through their enhanced thermal mass result in noise reduction. On the other hand, the utilization of larger temperature sensors increases the response time by a factor of 5.

The required responsiveness of the sensors depends on the specific application within the HVAC system. This paper summarizes the first results of an ongoing project, whose goal was to develop cost-effective, non-invasive sensors for monitoring and analysis of existing HVAC systems. The time constants for transients in such systems (like switching of pumps or changing of valve openings) are in the order of minutes. Sudden changes are difficult to achieve because of the hydraulic resistance and are depreciated, anyway, to avoid hydraulic shocks that cause excessive noise and can damage the system. Therefore, the measured responsiveness of both sensor prototypes is sufficient.

The influence of the distinct $1/f^{\alpha }$-noise observed for both prototypes can be reduced by signal averaging, where the applicable averaging period depends on the measured response times. Therefore, the averaging effects of bulky Pt100 elements are not a decisive benefit. It seems more advantageous to realize faster reacting sensors based on thin Pt100 elements and, if necessary, to reduce the elevated noise level by appropriate signal averaging. As a compromise, two or more small self-adhesive Pt100 elements could be distributed over the circumference to benefit from the spatial averaging effects and still obtain a sensor with short response times.

Owing to the temperature dependence of the thermal resistance and the high overall amplification, the output signal depends on the fluid temperature regardless of the specific operating mode. Constant excess temperature mode and constant heating power mode require complex controller circuits but will not significantly reduce the temperature dependence of the output signal. Therefore, the constant current mode with simplified sensor electronics was applied. The sensor prototypes were tested in a volume flow range between 1.5 L/min and 10 L/min. By appropriate adjustment of the sensor electronics, lower or higher flow range can also be achieved. The general drawback, however, is the saturation of the output characteristic with increasing flow velocity.

The temperature dependence of the output signal can be taken into account by adjusting the offset of the second amplifier. The corresponding correction function must be acquired experimentally. The microcontroller measures the upstream fluid temperature and applies the correction function to calculate and set the amplifier’s offset. The feasibility of this concept was tested during long-term measurements. Despite low resolution of the microcontroller’s ADC (only 10 bit), an overall relative error of ± 3% could be achieved. Moreover, during the measurement period with a constant fluid temperature, the relative error drops to only ±1%.

All measurements and characterizations were performed at constant room temperature. However, as the sensor is well isolated from the ambient (see Figure 2d–f), a significant influence of the ambient temperature on the output signal is not expected. The envisaged locations of the sensors are rooms with low variations of ambient temperature (e.g., basements). The investigation of this dependence will be a topic of future characterizations. If needed, the corrections of the output signal similar to one presented in Section 4 can be applied.
